# Intraperitoneal onlay mesh repair as rescue therapy for recurrent inguinal hernia following robotic transabdominal preperitoneal repair: a case report

**DOI:** 10.1093/jscr/rjaf523

**Published:** 2025-07-22

**Authors:** Anupam K Gupta

**Affiliations:** General and Minimally Invasive Surgery SSM Health, Mt. Vernon, IL, United States

**Keywords:** recurrent inguinal hernia, robotic TAPP repair, intraperitoneal onlay mesh (IPOM), peritoneal closure failure, morbid obesity, barbed sutures

## Abstract

Inguinal hernia is one of the most common procedures performed using a robotic transabdominal preperitoneal (TAPP) approach. Recurrence can occur due to failed reperitonealization. A breach in the peritoneum exposing mesh to bowel can lead to severe complications, requiring surgical correction. We describe a case where, despite repeated closure attempts, the peritoneum could not be approximated. An intraperitoneal onlay mesh was used as rescue therapy in a morbidly obese, diabetic female with multiple comorbidities on Plavix.

## Introduction

Robotic transabdominal preperitoneal (TAPP) inguinal hernia repair is widely used and associated with a low recurrence rate (<1%) [1]. It is especially helpful in bilateral hernias and obese patients [2]. The technique involves lowering the peritoneum, dissecting the hernia sac, and placing mesh over the myopectineal orifice [1, 2]. Early recurrence may result from inadequate dissection, mesh fixation failure, missed hernias, or improper peritoneal closure. Peritoneal closure must be tension-free to avoid complications [3]. Barbed sutures (V-Loc) in a small-bite mattress configuration are standard [4]. We report a case of repeated peritoneal closure failure in a morbidly obese diabetic patient, managed with intraperitoneal onlay mesh (IPOM) as rescue therapy.

## Case report

A 71-year-old woman (BMI 44 kg/m^2^) with insulin-dependent diabetes (HbA1c 7.6), hypertension, hypothyroidism, and depression presented with right lower quadrant pain. Due to obesity, clinical exam was inconclusive. Computed tomography (CT) revealed bilateral inguinal hernias: an incarcerated right and a recurrent left. She had a prior laparoscopic left inguinal hernia repair. She was on insulin, aspirin, Plavix, and other medications; Plavix was held preoperatively.

Robotic bilateral TAPP was performed. The peritoneum was dissected between anterior superior iliac spines. A large Bard 3DMax mesh (4×6 in) was placed bilaterally and anchored using absorbable tackers. Peritoneal closure was performed in two layers using V-Loc barbed sutures in a tension-free mattress configuration. The patient was discharged the same day.

**Figure 1:**
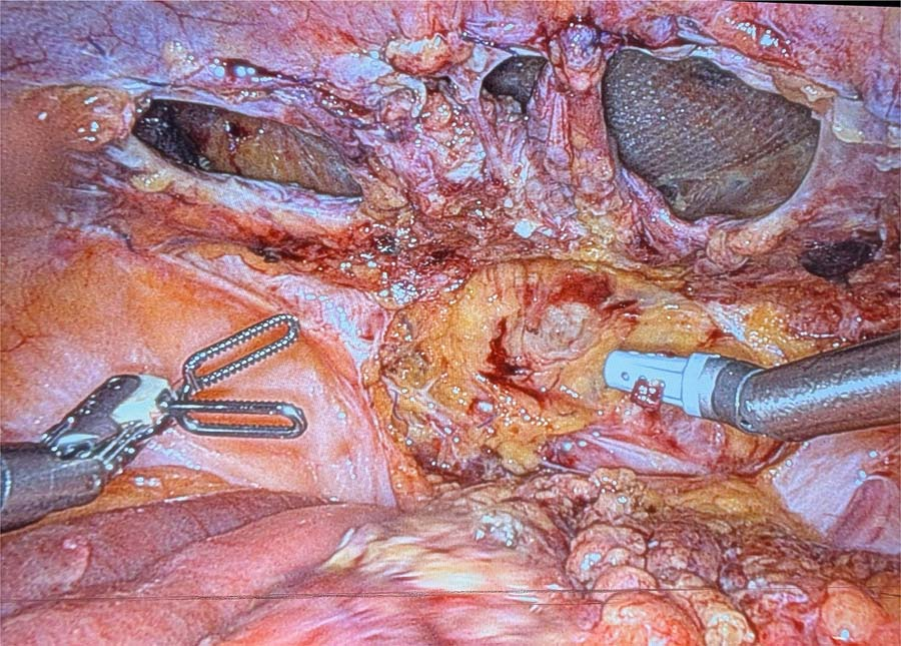
Recurrence on postoperative day 4 image showing multiple defects in the peritoneal closure.

On postoperative day 4, she returned with nausea, vomiting, and obstipation. CT showed an infraumbilical hernia. Re-exploration revealed peritoneal defects with minimal adhesions (Fig. 1). The mesh was intact, and bowel loops were easily reduced. Closure was repeated with V-Loc in two layers. She was discharged on postoperative day 2.

**Figure 2:**
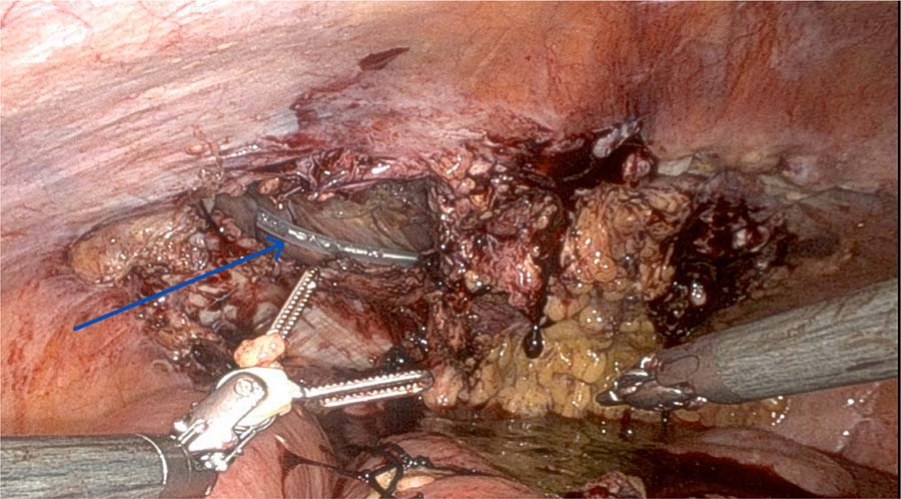
Recurrence on post-operative day 24 showing a significant peritoneal defect on the left side.

**Figure 3:**
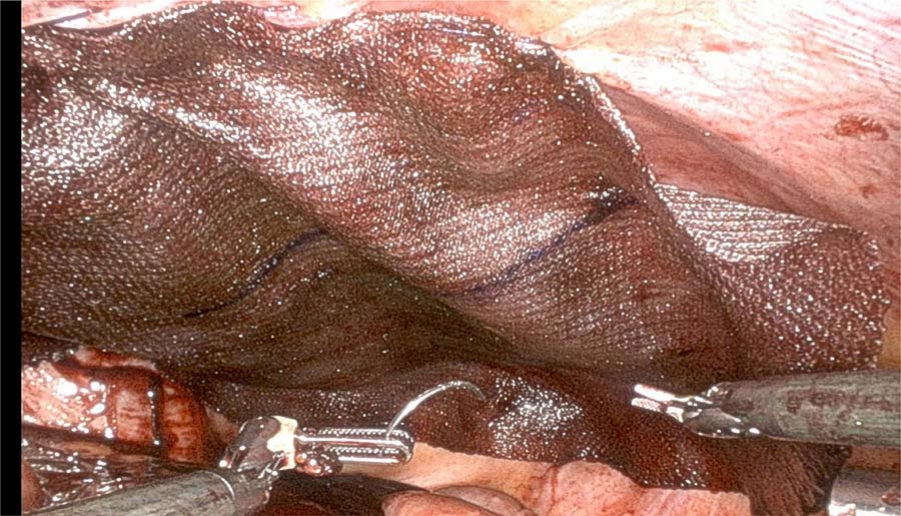
Repair with use of Ventralex ST mesh 10 × 8 in as intraperitoneal onlay fashion.

On day 24, she presented with abdominal discomfort. CT revealed recurrent right inguinal hernia with bowel herniation near the mesh. Robotic reoperation showed dense adhesions at prior suture sites. A 4×6 cm peritoneal defect could not be closed (Fig. 2). No bowel injury or mesh infection was noted. A 10 × 8 in Ventralight ST mesh was placed intraperitoneally (Fig. 3). Follow-up CT confirmed successful repair with no recurrence.

## Discussion

This case also highlights a practical solution when conventional methods are exhausted. While IPOM is typically reserved for ventral hernias, its use in the groin region, though unconventional, provided durable coverage without additional tissue trauma. In highly scarred or friable fields, IPOM may help avoid further risk of recurrence. Such decision-making reinforces the importance of surgical adaptability and patient-specific planning [5].

Reperitonealization is a vital step in the TAPP approach. Failure to close the peritoneum may expose the mesh to visceral contents, elevating the risk of adhesion formation, erosion, fistula, and obstruction. While barbed sutures are widely used for their knotless advantages and efficiency, they may contribute to suture-line adhesions or tension-induced failures, especially when tissue integrity is compromised. Surgeons must weigh the benefits of speed against the potential for long-term complications [6, 7].

Robotic TAPP repair remains a cornerstone in modern hernia surgery, providing surgeons with a high level of precision and reducing recovery time for patients. Nevertheless, as illustrated by this case, success still depends heavily on individual patient anatomy, comorbid conditions, and technical nuances. In patients with morbid obesity, anatomical distortion and limited workspace increase the likelihood of peritoneal closure challenges and recurrence. Additionally, delayed tissue healing associated with diabetes further complicates the postoperative course [8, 9].

Early recurrence may be linked to technical errors or patient factors like diabetes, obesity, and tissue quality [6, 7]. This case highlights diagnostic challenges in obese patients, where CT is essential [5]. At reoperation, scarring and adhesions made primary closure impossible. IPOM provided a feasible solution. Though not routine for inguinal hernias, IPOM may be considered in select salvage scenarios [10–13].

Robotic TAPP repair is effective and offers advantages in morbidly obese patients [14, 15]. However, obesity increases the risk of recurrence, and anticoagulants add complexity [10–13]. In this case, despite proper mesh placement and technique, peritoneal closure failed twice. Barbed sutures, though standard, may cause adhesions or bowel obstruction [8, 9, 16].

## Conclusion

In morbidly obese, comorbid patients, recurrent hernia following robotic TAPP can result from peritoneal closure failure. When reperitonealization is not possible, intraperitoneal onlay mesh may serve as effective rescue therapy.
